# Inconvenience of Healing Wounds by the First Intention

**Published:** 1800-01

**Authors:** G. Nesse Hill

**Affiliations:** Surgeon. Chester


GENTLEMEN,

SHOULD the following remarks on an important branch of the modern Practice of Surgery, be found deſerving a place in your uſeful Publication, by inſerting them at your convenience, you will oblige, Your humble ſervant,

*Cheſter, Dec.* 12, 1799. G. NESSE HILL, Surgeon.

---

THE numerous and great improvements which the practice of ſurgery has derived from the diligent labours of modern profeſſors, is highly honourable to all who have in any degree contributed to the increaſe of knowledge, ſo conducive to the ſafety and comfort of mankind; hence it will be readily granted, that however ſmall the mite which is added to the general ſtock, if it prove truly an acquiſition, its minuteneſs will not cauſe it to be undervalued. Surgical books and lecturers have, of late years, ſtrongly recommended the practice of healing wounds of a certain deſcription, by what is appoſitely called the firſt intention; of a certain deſcription, I repeat, becauſe I believe no practitioner in the preſent day recommends this mode, but in wounds which happen to be inflicted by clean-cutting inſtruments upon healthy ſubjects, and in favourable ſituations, no comminution or conſiderable laceration having taken place; or, laſtly, after ſome ſurgical operation of a kind favourable for the practice. However agreeable this method may generally be to the patient, and how much ſoever it may ſeem to evince the dexterity of the ſurgeon, yet it is a certain fact, the conſequences are not always pleaſant to the former, or creditable to the latter. Several caſes have fallen under my obſervation, where I have no heſitation in ſaying, it has proved injurious. Amongſt others the following ſhort hiſtory will point out a caution to practitioners, not unworthy the attention of the warmeſt advocates for this facile method of healing wounds.

A reſpectable druggiſt in London was preſſing a cork into a bottle of Daffy's Elixir with his right hand, the neck of which breaking, he received a deep wound thereby in a longitudinal direction, juſt below the firſt joint of the thumb; having no reaſon to imagine any part of the glaſs remained in the wound, and moving the part juſt the ſame as before the accident, he deſired one of the ſhop-men to apply ſome lint, dipped ped in the Tinct. Benzoës c. over the lips of the wound, previously held tight together, and bind all up with adhesive plaister; the subsequent day, feeling some pain, he shewed it to his surgeon, who on seeing all so smooth, and the parts but little swelled, said the dressings had better remain as they were, and perhaps the wound would heal by the first intention; this advice was taken, and accordingly it did so heal; but he has never since been capable of bending his thumb, inwards or downwards, without the assistance of his other hand; the extensor muscles keep it constantly strait out, the tendon of the flexor pollicis muscle appearing to be firmly united to the bone, at the place where the wound was inflicted; thus the proper action of so important a part is in a great measure destroyed.

In the relation of a case in surgery, as in the description of morbid appearances of dissected bodies, improvement in our art being the primary object, every man will, and most certainly ought to judge for himself: on examining the above thumb, it appeared to me worthy of attention, as confirming me in my former opinion, that due care is not always taken to discriminate between situations of the body favourable to healing wounds by the first intention, and healing by suppuration, &c. In the former mode, divided parts are always more or less condensed together by adhesive plaister, compress, or bandage, and consigned to rest so long in one posture to favour their speedy re-union, that coalition follows where it never ought; and Nature, if I may use the expression, taking it for granted they are never to be used, yields to their becoming immoveable; and thus, an important flexible part becomes, ever after, rigid and inflexible, or its proper function is at least greatly impeded. The effects of even the slight degree of inflammation necessary to heal a wound in an healthy subject, by the first intention, is not strictly confined to the spot we want to unite, but affects the neighbouring parts to a proportionate extent; sometimes in a greater, sometimes in a less degree, as every day's practice sufficiently evinces; and it is perhaps equally well known, that pressure considerably increases the action of the absorbent system.

Inasmuch therefore as the perfection of good surgery depends upon as complete a restoration of deranged parts, whether by art or accident, to their former functions and usefulness as possible; it is incumbent upon us, to attend strictly to the situation of wounds, before we determine on the mode of attempting their cure; for want of this precaution in wounds of the tendons, the arteries, veins, &c. many serious consequences have followed; nor is the injunction to be less regarded after surgical operations, particularly amputations, or thofe performed in the vicinity of an articulation, or of the fphincters of the anus, or bladder. I remember two cafes of perfons cut for the ftone, where the attempt fucceeded, of healing the divided parts by the firft intention; but both fubjects were ever after grievoufly troubled with an incontinence of urine.

---

### *Hints on the Medicinal Effect of Living with Cows, in Pulmonary Confumption. Communicated by* Mr. Blair, Surgeon of the Lock Hofpital and Finfbury Difpenfary, &c.

---

